# Pedestrian counting estimation based on fractal dimension

**DOI:** 10.1016/j.heliyon.2019.e01449

**Published:** 2019-04-08

**Authors:** Andrés C. Jiménez, John Anzola, Alexander Jimenez-Triana

**Affiliations:** a Department of Electronic Engineering, Fundación Universitaria Los Libertadores, Carrera 16 No. 63 A – 68, Bogotá, Colombia; b Department of Control Engineering, Universidad Distrital Francisco José de Caldas, Cll 74 Sur No. 68A - 20, Bogotá, Colombia

**Keywords:** Computer science

## Abstract

Counting the number of pedestrians in urban environments has become an area of interest over the past few years. Its applications include studies to control vehicular traffic lights, urban planning, market studies, and detection of abnormal behaviors. However, these tasks require the use of intelligent algorithms of high computational demand that need to be trained in the environment being studied. This article presents a novel method to estimate pedestrian flow in uncontrolled environments by using the fractal dimension measured through the box-counting algorithm, which does not require the use of image pre-processing and intelligent algorithms. Four scenarios were used to validate the method presented in this article, of which the last scene was a low-light surveillance video, showing experimental results with a mean relative error of 4.92% when counting pedestrians. After comparing the results with other techniques that depend on intelligent algorithms, we can confirm that this method achieves improved performance in the estimation of pedestrian traffic.

## 1 Introduction

Video analytics is a technology capable of extracting information and behavior patterns, and performing object identification and classification, among its many other capabilities in commercial, security, government, transport, surveillance and monitoring applications across different sectors.

One of the features of Video Analytics' algorithms is their easy implementation in embedded systems. Video Analytics can be installed on conventional computers but also on development platforms such as the Raspberry Pi and other hardware with integrated DSPs (Digital Signal Processors) used in IP cameras and other devices.

When Video Analytics' algorithms are integrated with video, they are referred to as Video Software Systems [1]. These systems perform tasks that use statistical techniques and machine learning to extract and identify individual persons, track them, and compute the number of pedestrians automatically in high-traffic areas, with direct applications in real-time processing systems [2], [3], [4], [5], [6].

The data obtained through pedestrian counting can be, in turn, directly applied to pedestrian flow detection, surveillance systems for tracking and detection of inappropriate behavior in low-density areas, urban planning, marketing studies in shopping malls, and critical area analysis in evacuations [7], [8].

Pedestrian counting is a sub-area of detection and surveillance, an activity that includes tasks of tracking, searching, detection, and classification of people's behavior in video analytics. These tasks have been the focus research over the past few years due to population growth in cities [7]. The settling and attendance of people in some areas have resulted in an uptake in surveillance systems, which are comprised of one or several human operators who are in charge of visually monitoring a camera system. There is an intrinsic inefficiency area in this system that becomes evident when the number of cameras exceeds the number of human operators, as a result of the lack of sufficient visual coverage by the operators. For this reason, it becomes evident that there is a need for an intelligent image-processing system that is tasked with detecting possible anomalies and support decision-making in traffic control, without resorting to an increase in the number of human operators [5], [9].

In the study of environments with the presence of crowds to estimate the number of pedestrians, it is important to understand if the scene being processed is structured or unstructured. In a structured scene, the crowd moves coherently, keeping a movement pattern that is constant in time. An example of this is a security video of a pedestrian crossing, where people are forced to follow a path to cross the street. In an unstructured scene, crowds do not follow any specific behavior pattern, which means that people move in any direction and create multiple paths that need to be processed [10]. To estimate pedestrian counts in any of these types of scenes, several categories have been used, among which we can mention intelligent algorithm detection [11], [12], segmentation detection [13], and cluster detection [14].

Some of the salient features of the intelligent algorithms' category are the ability to detect pedestrians in structured and unstructured scenarios with a very low error rate, due to avoiding the issue of occlusion among different persons and objects. In the case of [15] the intelligent algorithm uses a “visual regressor” that diminishes the impact of pedestrian occlusion and dynamic change in the distribution of pedestrians in the environment, for which its implementation requires a high level of computational capacity. This trait is also shared by clustering algorithms, which require a high capture frame rate to track changes in images, as is the case of the paper by Zhou et al. in [2] where each pedestrian in the image is presented as an agent that tries to represent their starting point and destination. These two types of algorithms are intended for behavior study and prediction.

Conversely, Viola et al. [16], defines an approach that uses machine learning by using cascading classifiers, which allows eliminating areas that do not contain objects matching the domain learned by the system. However, when this approach is applied to person-detection in an uncontrolled environment, it presents problems with occlusion between pedestrians. To improve person-detection with occlusions, Zhang et al. [17] use a Support Vector Machine (SVM) Histograms of Oriented Gradient (HOG) descriptors, but this approach requires significant computational power to complete the classifications of pedestrians in the scene. Recently, deep learning techniques such as Convolutional Neuronal Networks (CNN) and MobilNets have been applied in the detection of pedestrians and object classification in scenes [18], [19].

While segmentation algorithms requires a low level of computational capacity, they present occlusion issues caused by the different objects in the environment and by lighting problems [20]. These issues can be reduced if the images are modeled as objects with self-similar, geometrical or fractal structure, this modeling is known as fractal analysis that uses the fractal dimension as the measure. Anam et al. [21] apply this model, where it helps reduce lighting errors in X-ray images. Fractal analysis can be applied directly on scenes for person count estimation, as these scenarios—and most natural objects and scenes—are full of complex forms that cannot be easily modeled by Euclidian geometry. Fractal geometry can be used to characterize and model objects with irregular outlines such as mountains, clouds, textures, trees, among others [22].

In the environments analyzed in pedestrian counting, irregularities are produced by the dynamics of motion and the number of people crossing a particular area. If the captured images are represented in grayscale, their level of irregularity can be measured through the fractal dimension, a dimension that is between the second and third dimensions. If the surface of the objects being analyzed has a smooth outline, their fractal dimension will be closer to the second dimension. Alternatively, when outlines are rough or irregular, the fractal dimension of the objects will be closer to the third dimension [23].

In this article, we develop a novel method to estimate pedestrian count that is based on calculating the fractal dimension of images in structured or unstructured scenes, by using segmentation algorithms. To validate the proposed method, a streaming video was captured from a surveillance camera, processing the level of irregularity of the environment, which changes with the number of pedestrians crossing the scene. This enabled to obtain an estimative pedestrian count, without being affected by color shift or low lighting.

To measure the fractal dimension, it applied the box-counting method. This method was selected on account of its simplicity, low computational cost and autonomy, which allows its implementation on any image and resulted in an outstanding level of accuracy in estimating the fractal dimension, as long as the number of pixels on the *x* axis is equal to the number of pixels on the *y* axis [24], [25], [26]. Experimental results are compared with techniques using intelligent algorithms, such as CNN and MobilNets.

The proposed method stands apart for two main contributions. The first one is that it reduces the computational requirements because it does not require to process each individual to estimate the pedestrian count. This means that it does not require pre-processing, classification, tracking or learning techniques. The second, is that by not depending on a learning system, can be easily applied in any environment.

This article is organized as follows. The box-counting algorithm used to estimate the fractal dimension is explained in section 2. Section 3 validates the use of fractal dimension through box counting to estimate pedestrian count in four different environments. Section 4 presents the results of applying the differential box-counting method to estimate the number of pedestrians in an uncontrolled environment. Finally, section 5 presents the conclusions.

## 2 Theory/calculation

To discuss the fractal dimension, we first need to describe the Hausdorff dimension. Given a metric space (X,d), where *X* is a defined set with a measurement *d*, and a closed ball defined in metric space B(x,r):={∀y∈X,d(x,y)<r}, and defining a cover $C=\left\{U_{\alpha }:\alpha \in X\right\}$ that contains a set of open balls, we can estimate that the Hausdorff dimension D of a subset X of the Euclidian space can be determined by counting $G_{r}$ balls contained in the cover with a radius $r=\varepsilon \in R^{+}$, the Hausdorff dimension *D* can be expressed in (1).

(1) $$D=\lim_{x\to \infty }\left(\frac{\log G_{r}}{\log\frac{1}{r}}\right)$$

However, although this measure can express with great precision the fractal dimension of objects, it has the great disadvantage of not being able to be calculated through computation. For this reason, several authors highlight the importance of working with the Box Counting Dimension (BCD) to calculate the fractal dimension.

This measurement accurately expresses the fractal dimension of objects, but it has the great disadvantage of only being able to be calculated through computation in cases where the fractal is deterministic. For this reason, several authors point out the importance of working with BCD, and in particular with the DBC technique to calculate the fractal dimension in non-uniform images [24], [26].

**Figure 1:**
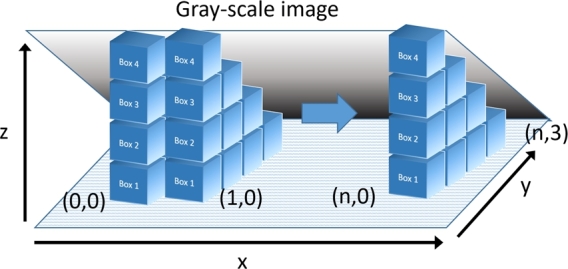
Measurement using the Differential Box Counting Method over a 2-D gray-scale image.

Two-dimensional images can be considered as three-dimensional when they are represented by M×N×H, where *M* is the number of pixels in the *x* axis, and *N* is the number of pixels in the *y* axis, and height *H* is the intensity in grayscale of a specific pixel, that can be calculated as H=f(x,y):{x∈M,y∈N}. The DBC technique consists in having a symmetrical image where pixels in the *x* axis must be equal to the pixels on the *y* axis i.e. (M=N). Ensuring that the images are symmetrical, we can generate blocks of m×n×h size, where $\left\{m,h\in R:\frac{M}{2}\geq m>1,1\leq h<256\right\}$ if the image is *8-bit*. After the generation of the block we must count how many blocks are needed to cover the complete image, using as measure (2), given *r* the radius of the ball from (1), or in this case, the scale factor represented by the measure *m* of the cube. Figure 1 shows how the blocks are generated from a grayscale image modeled in 3-D.

(2) $$g_{r}\left(i,j\right)=\max\left(\frac{f\left(x\pm i,y\pm j\right)}{r}\right)-\min\left(\frac{f\left(x\pm i,y\pm j\right)}{r}\right)+1$$

**Figure 2:**
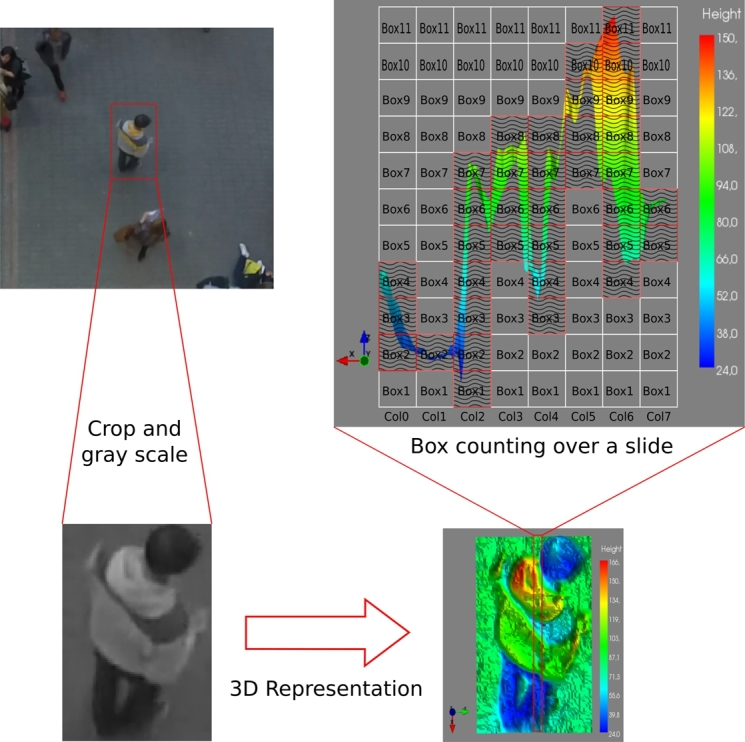
Differential Box-Counting method in a two-dimensional image.

The total block count can be estimated from the sum $G_{r}=\sum_{i,j}g_{r}\left(i,j\right)$, which when replaced with the measurement *r* of the cube in equation (1) estimates the approximate fractal dimension, e.g. Figure 2 shows the process of image capture, from which a square section is extracted and converted to grayscale and then visualized as a 3-D image, extracting a slice of the image to determine its approximate fractal dimension. The slice is visualized on the *x* and *y* axis only for the purposes of this explanation. This means that only the width and length of the images are visualized as shown in the upper right area of Figure 2. The slice is processed by box counting by using blocks of 14px×14px×14px, making a total of 8 columns. The number of boxes in the first column is estimated by $g_{r}\left(col_{0}\right)=4-2+1=3$, the second column doesn't show significant changes in relief is only counted by one box from $g_{r}\left(col_{1}\right)=2-2+1=1$. In the third column, that presents a drastic change in the image, the number of boxes in that area is $g_{r}\left(col_{2}\right)=7-1+1=7$. The previous example shows a problem of scale in the measurements of the cube. This shift can be covered with just 6 cubes. To solve this problem, Li et al. [25] proposes that the minimum and maximum levels of the cube in f(i,j) be approximated to the highest and closest integer number, as shown in (3). This is, if the result is 3.1 cubes in the maximum value, the value should be approximated to 4.

(3) $$g_{r}\left(i,j\right)=\max\left(ceil\left(\frac{f\left(x\pm i,y\pm j\right)}{r}\right)\right)-\min\left(ceil\left(\frac{f\left(x\pm i,y\pm j\right)}{r}\right)\right)+1$$

## 3 Calculation

To validate the pedestrian count estimate using the DBC technique in images, we use four controlled environments. The image background is constant, there are different geometric objects and people, and images with a resolution of 500px×500px are used, obtaining the DBC in each processed image with blocks of 4px×4px×4px, to estimate their fractal dimension.

**Figure 3:**
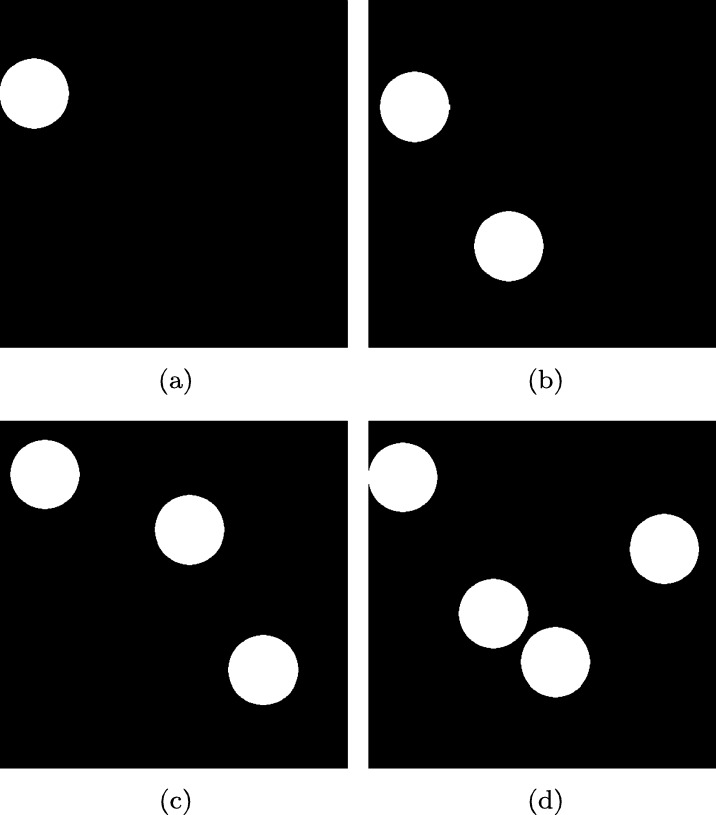
Samples from the first environment with **(a)** one ball, **(b)** two balls, **(c)** three balls, and **(d)** four balls.

**Figure 4:**
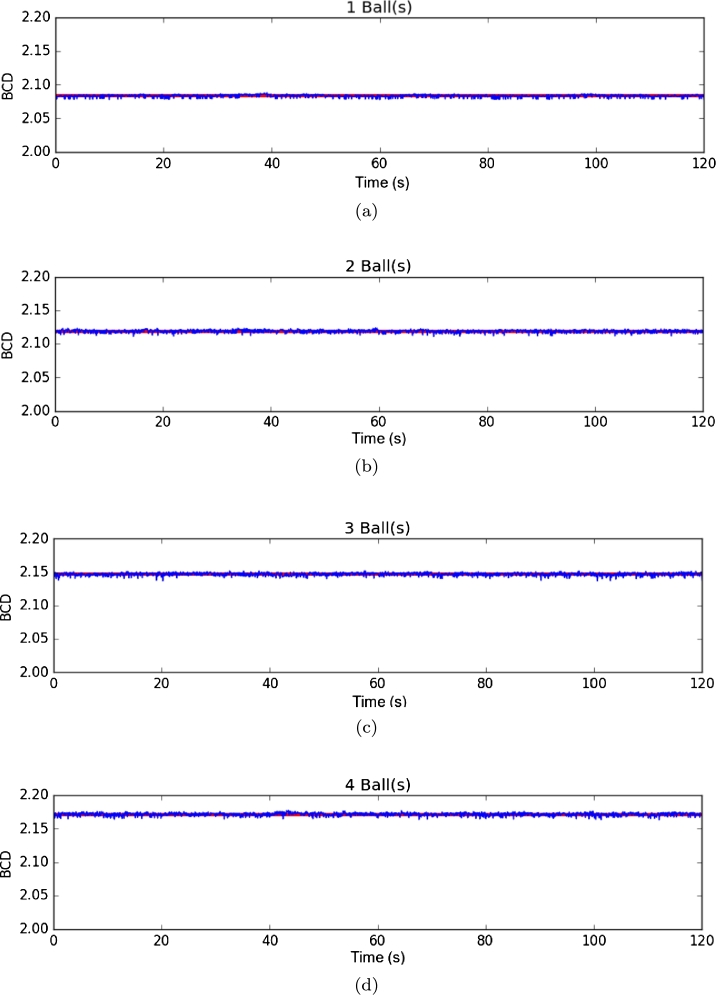
Results from the first environment. **(a)** One ball, **(b)** two balls, **(c)** three balls, and **(d)** four balls.

**Figure 5:**
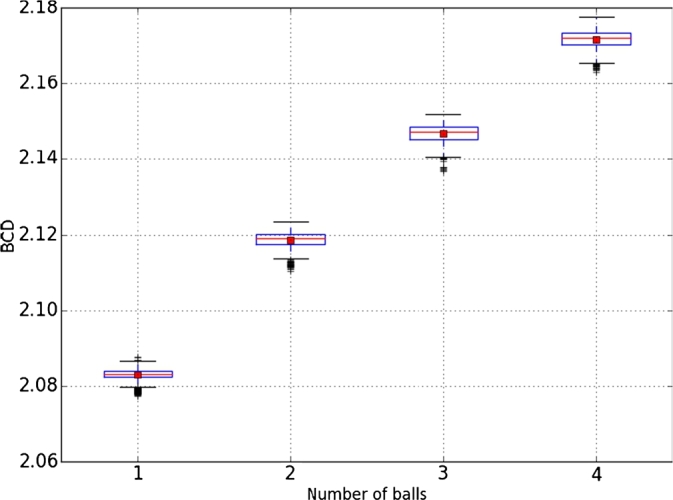
Box plot from the first environment.

The first environment used a dark gray background, and pedestrians were represented with white spheres in motion. The fractal dimension of the scene was calculated in 4 experiments, 2 minutes long each, with a different number of spheres Figure 3. These four experiments were performed without any image filtering or processing technique. Results are shown in Figure 4 and Figure 5.

To verify the performance of DBC in an image versus the number of objects in the environment, we analyzed the median and standard deviation in each of them, which results in Table 1, where the change of fractal dimension by box counting is shown when the number of spheres in the scene changes.

**Table 1:** Statistical results from the DBC results on the first environment.

Number of balls	DBC Mean	Standard deviation
1	2.08309668706	0.00182956888
2	2.11864130332	0.00213365802
3	2.14691700568	0.00231306910
4	2.17176830455	0.00234066708

**Figure 6:**
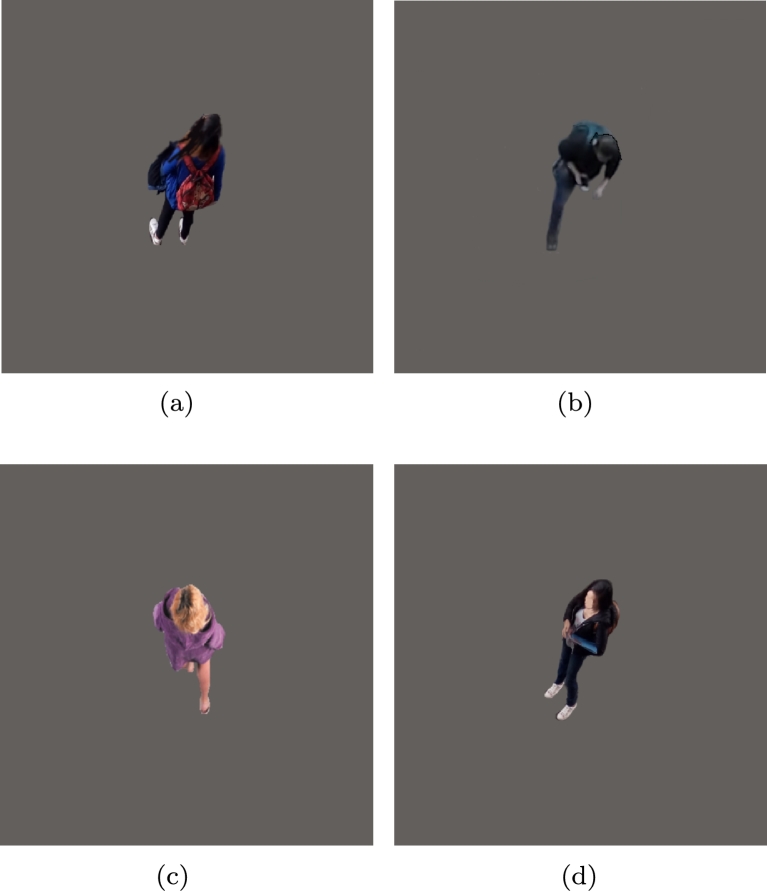
Four samples from the second environment with one pedestrian. **(a)** Woman with dark dress, **(b)** man with dark dress, **(c)** woman with a purple light dress, and **(d)** woman with dark dress.

In the second environment, there was a single person over a gray background, simulating the color of a sidewalk. The experiment was performed with a sample of 10 different persons, as shown in Figure 6.

**Figure 7:**
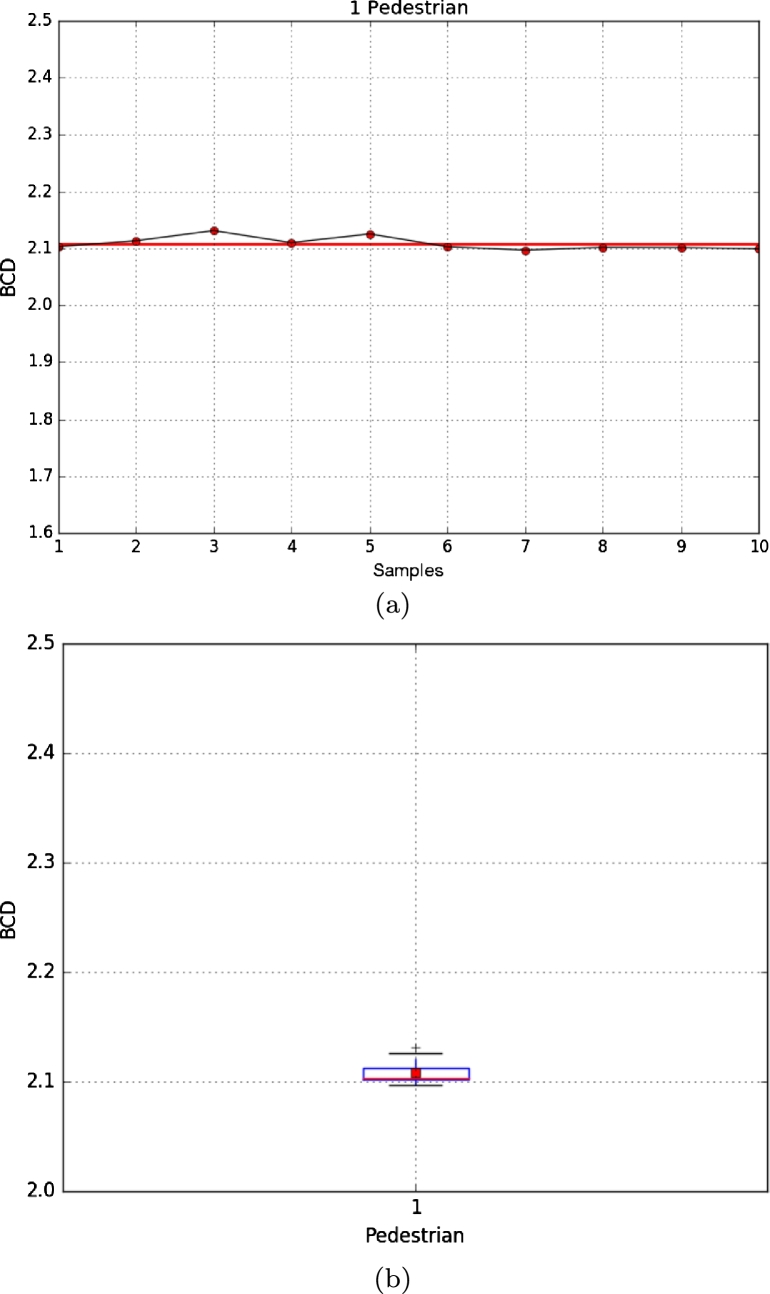
Results from the second environment with one pedestrian, **(a)** results obtained with 7 different persons, **(b)** box plot of the second experiment.

As in the first environment, the DBC of each image was calculated, obtaining a median μ=2.10872751712 and a standard deviation σ=0.0116160143136, as shown in Figure 7. This resulted in a low difference among samples, showing that DBC can estimate the number of pedestrians in an environment regardless of the type of clothing worn by pedestrians in the image.

**Figure 8:**
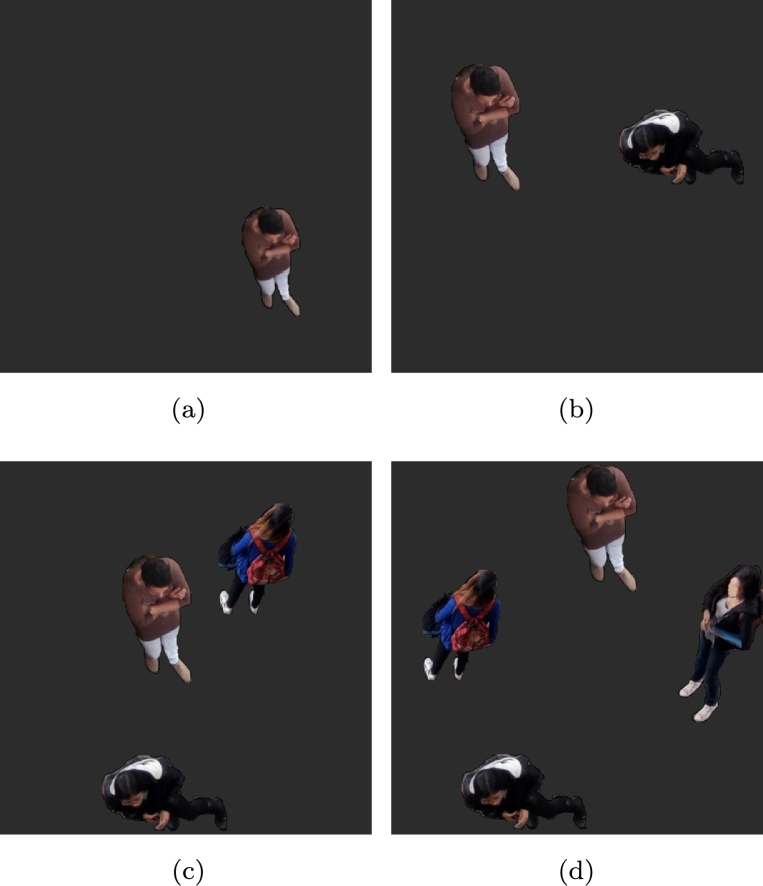
Four samples from the third environment, with four pedestrians. **(a)** One pedestrian, **(b)** two pedestrians, **(c)** three pedestrians, and **(d)** four pedestrians.

Once the experiments in the second environment validated that DBC produces different incremental measurements in relation to the number of people in the image, we conducted the last test in an environment where, regardless of the number of people present in the image, DBC allowed to estimate the number of people in the environment. This test consisted of 4 experiments with 2 minute videos, with different numbers of people (Figure 8), starting from 1 pedestrian to 4 pedestrians.

**Figure 9:**
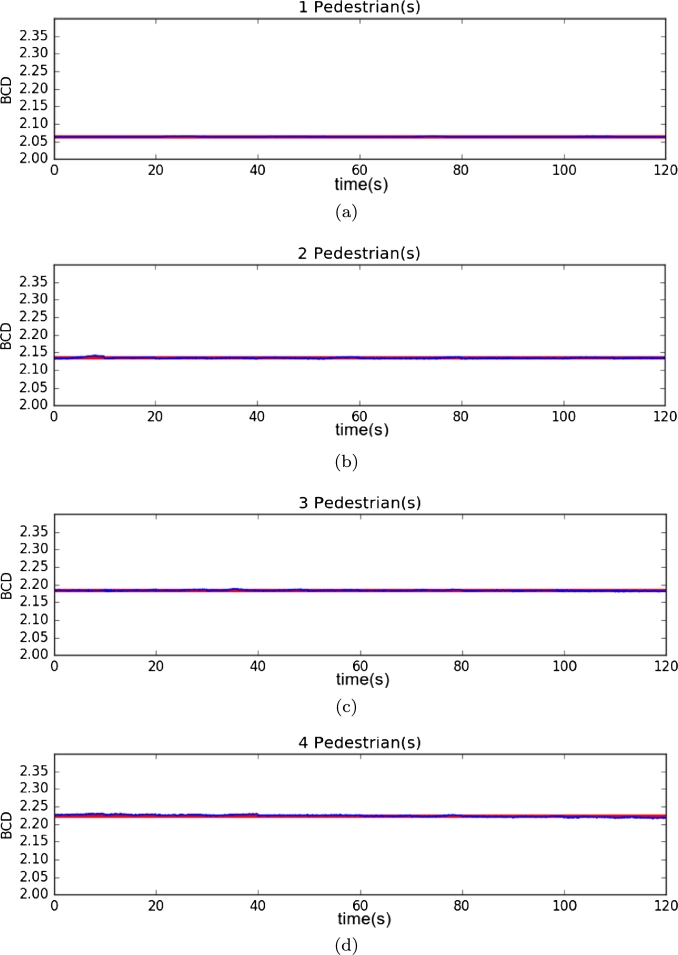
Results of the third environment.**(a)** One pedestrian, **(b)** two pedestrians, **(c)** three pedestrians, and **(d)** four pedestrians.

**Figure 10:**
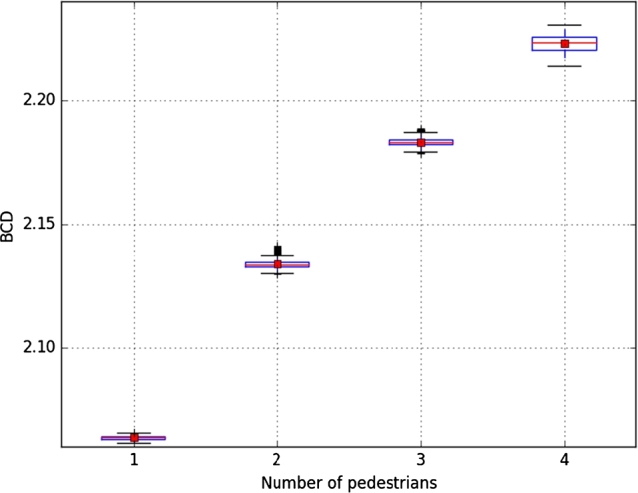
Box plot of the third environment.

Table 2 and Figure 9 show the results of the four experiments, showing that DBC can be used to estimate pedestrian count by calculating the changes in relief in the complete image when there is a different number of pedestrians in the scene. Figure 10 shows the distribution of values.

**Table 2:** Statistical results from the DBC results on the third environment.

Number of pedestrians	Median	Variance
1	2.06367077455	0.0007112044120
2	2.13385732661	0.0016774378554
3	2.18309237163	0.0014926512587
4	2.22316542849	0.0031835984765

## 4 Results and discussion

Up to this point, we used images with controlled backgrounds to estimate the number of pedestrians. This section consists of DBC processing in uncontrolled videos, this is, where the background, the number of people, and lighting change dynamically in time. As in section 3, we worked with videos with a resolution of 500px×500px, with a duration of 6 minutes. DBC processing used blocks of 4px×4px×4px. The dataset could be download from [27].

**Figure 11:**
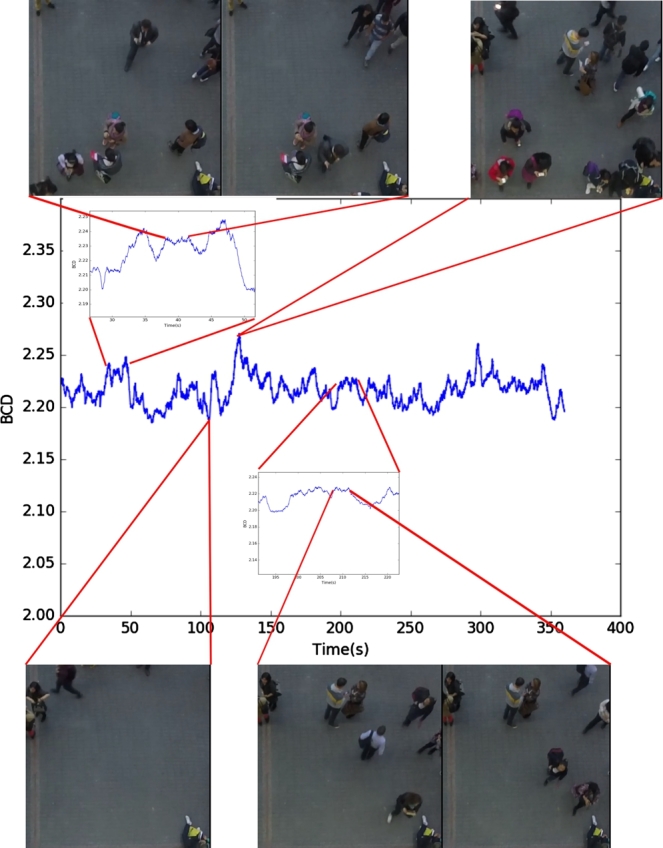
Calculation of the DBC in the fourth environment.

Figure 11 shows the DBC calculation for the complete video, in 4 zones. The first zone goes from 40 to 44 seconds, with a constant number of pedestrians equal to 8. The second zone is at the 106 seconds mark, with only 3 pedestrians, which was the lowest number of pedestrians in the video. The third zone is at 128 seconds, showing a total of 16 pedestrians, which was the highest number of pedestrians in the video. Finally, the fourth zone is from 208 to 212 seconds of the video, where the number of people remained constant at a value of 7.

To begin estimating the number of people in the scene, we took samples every 2 seconds, counting the number of people manually and then calculating the fractal dimension by box counting and generating a ratio of people to DBC. With this information, we separated the samples by the number of people to calculate the median and standard deviation, as shown in Table 3.

**Table 3:** Relation between number of pedestrians and the fractal dimension using the DBC method.

Number of pedestrians	DBC Mean	Standard deviation
1	–	–
2	–	–
3	2.1890760838	0
4	2.1982309295	0.009035051
5	2.2000230109	0.004381833
6	2.2077734657	0.007151711
7	2.2111005227	0.005706795
8	2.2212754772	0.00874453
9	2.2216404012	0.005436004
10	2.2284829039	0.005764352
11	2.2286388561	0.002292419
12	2.2347186296	0.00339837
13	2.2427816279	0.002359135
14	2.2554608746	0.002992223
15	–	–
16	2.2663613482	0

Equation (4) shows the function generated by applying the linear regression with the values of Table 3, where *x* is the fractal dimension and f(x) is the estimated number of pedestrians. We must take into account that the domain of the function is real numbers, but the codomain is the positive integer numbers. As it represents a number of people $f:R\to Z^{+}\cup \left\{0\right\}$, it is necessary to use the function round(.) to approximate the number to the nearest integer number.

(4) f(x)=round(175.1x−380.18)

Applying the data of Table 3 in (4), we find the relative error between the estimate pedestrian count and the number of pedestrians counted manually (Table 4). The average relative error is 4.92%. The error is incremented by the inclusion of elements that changes the fractal dimension, also the use of integer numbers instead of real numbers to represent pedestrians that are partially in the image, i.e. they are entering or leaving the processing area affects the estimation. However, the results show a good estimate of the number of people in an uncontrolled environment without using processing techniques in the captured images.

**Table 4:** Relation between number of pedestrians and the fractal dimension using the DBC method.

Pedestrians	Hausdorff dimension	Estimated pedestrians	Relative error	Time (s)
3	2.189076084	3.127222	4.24%	0.0385
4	2.19823093	4.730236	18.26%	0.0500
5	2.200023011	5.044029	0.88%	0.0487
6	2.207773466	6.401134	6.69%	0.0392
7	2.211100523	6.983702	0.23%	0.0373
8	2.215695473	7.788277	2.65%	0.0401
9	2.221640401	8.829234	1.90%	0.0393
10	2.2274018	9.838055	1.62%	0.0372
11	2.229625491	10.22742	7.02%	0.0377
12	2.23471863	11.11923	7.34%	0.0361
13	2.242781628	12.53106	3.61%	0.0373
14	2.255460875	14.7512	5.37%	0.0375
16	2.266361348	16.65987	4.12%	0.0376

Average			4.92%	0.0397

### 4.1 Complementary evaluations

To validate the efficiency of the estimation presented in the present article, we repeated the experiment in an identical environment, selecting frames which contained a specific number of pedestrians. These frames were processed by applying the following methods independently: Haar Cascades for Human Detection [16], [17], Histograms of Oriented Gradients for Human Detection (HOG) [18], MobilNets [19], and Convolutional Neural Networks.

Table 5 presents the results of the experiments, showing that the method employing CNN presents a relative error of 16.19%, which is the smallest of all methods applied.

**Table 5:** Results using Haar Cascades for Human Detection, HOG, MobilNets and Convolutional Neural Networks.

Method		Haar Cascades		HOG		MovilNets		CNN	
Snapshot time (s) Figure 11	Pedestrians	Estimated	Relative error	Estimated	Relative error	Estimated	Relative error	Estimated	Relative error
106	3	0	100.00%	0	100.00%	3	0.00%	3	0.00%
8	4	2	50.00%	1	75.00%	3	25.00%	3	25.00%
20	5	2	60.00%	3	40.00%	5	0.00%	6	20.00%
12	6	0	100.00%	2	66.67%	6	0.00%	7	16.67%
1	7	3	57.14%	2	71.43%	7	0.00%	7	0.00%
34	8	3	62.50%	2	75.00%	5	37.50%	7	12.50%
48	9	4	55.56%	3	66.67%	8	11.11%	6	33.33%
46	10	3	70.00%	3	70.00%	9	10.00%	9	10.00%
178	11	4	63.64%	4	63.64%	5	54.55%	10	9.09%
124	12	2	83.33%	4	66.67%	10	16.67%	7	41.67%
130	13	3	76.92%	5	61.54%	10	23.08%	11	15.38%
126	14	4	71.43%	3	78.57%	9	35.71%	12	14.29%
128	16	3	81.25%	5	68.75%	15	6.25%	14	12.50%

Average relative error			71.67%		69.53%		16.91%		16.19%

However, this method does present a greater error rate compared to the method presented in this article. Additionally, it takes 432ms to process each frame, as shown in Table 6. On the other hand, the MobilNets and CNN methods were capable of identifying the exact position for each pedestrian while simultaneously classifying the objects detected in the frame, which prevents counting false positives like animals or bicycles.

**Table 6:** Average processing time per frame using Haar Cascades for Human Detection, HOG, MobilNets and Convolutional Neural Networks.

Method	Haar Cascades	HOG	MovilNets	CNN
Time(s)	0.022	0.115	0.088	0.432

## 5 Conclusion

This article presents a novel method to estimate the number of pedestrians, based on the differential box-counting method (DBC) to measure the fractal dimension in videos. The method proposed in this article was tested in four different environments, demonstrating its potential to autonomously estimate the number of pedestrians in a dynamic environment. This technique was implemented in uncontrolled environments, where the captured images were not pre-processed. This is a differentiating factor against current techniques, which require conditioning and segmentation techniques or using machine-learning algorithms to estimate the number of people in a scene. The mean relative error of this method in experiments was 4.92%.

It is important to point out that abrupt changes in light intensity affect the results obtained by using DBC in uncontrolled environments. However, the estimation of the number of pedestrians can be performed, as long as the parameters of the current environment are taken into account in the equation used for the approximation. In the case of this article, the function used was adjusted polynomials of the Taylor series. One limitation of the proposed method is that it cannot classify or track individual pedestrians in the scene.

For future work, this technique will be implemented in a wireless sensor network, to estimate the flow and direction of pedestrians. This would allow a study to analyze the behavior of movement in people in any environment, and to generate congestion alerts in evacuation scenarios.

## Declarations

### Author contribution statement

Andrés C. Jiménez: Conceived and designed the experiments; Performed the experiments; Wrote the paper.

John Anzola: Conceived and designed the experiments; Analyzed and interpreted the data; Wrote the paper.

Alexander Jimenez-Triana: Contributed reagents, materials, analysis tools or data; Wrote the paper.

### Funding statement

This research did not receive any specific grant from funding agencies in the public, commercial, or not-for-profit sectors.

### Competing interest statement

The authors declare no conflict of interest.

### Additional information

Data associated with this study has been deposited at https://github.com/acjimeneza/Hausdorff.
